# The ethics of resource allocation in translational genomic medicine

**DOI:** 10.1007/s12687-021-00517-4

**Published:** 2021-03-12

**Authors:** Christian Munthe

**Affiliations:** Department of Philosophy, Linguistics and Theory of Science, University of Gothenburg, Box 200, SE-40540 Gothenburg, Sweden

**Keywords:** Bioethics, Ethics, Health policy, Opportunity cost, Research ethics, Translational research

## Abstract

Two basic models of the rationale of translational genomic medicine (TGM)—the “Lab Assisting Clinic” (LAC) and the “Clinic Assisting Lab” (CAL) models—are distinguished, in order to address the ethics of allocating resources for TGM. The basic challenge of justifying such allocation is for TGM to demonstrate sufficient benefits to justify the opportunity cost of lost benefits in other areas of medicine or research. While suggested ethics frameworks for translational medicine build on clearly distinguishing these models, actual TGM typically blurs them. Due to lack of and difficulty in collecting evidence, prospects for justifying the LAC model currently seem poor, but this difficulty might be overcome by more research that tests the very concept of TGM. The CAL model aims to thus advance science, but is ridden by ethical hazard, undermining attempts at justification. This leaves the notion of running bona fide controlled trials of entire TGM concepts that have been justified from the perspective of clinical and research ethics (and approved by IRBs). It remains, however, an open question if the outcomes of such trials will demonstrate benefits that can justify the investment in TGM. To advance the prospect of such justification further, charting of the cost-benefit profile of TGM compared to alternative health investments would be helpful.

## Introduction

The notion of translational research as an agenda for enhancing research and clinical practice has had a quick uptake within genomic medicine (Cho et al. 2016; Schully and Koury 2014), linking closely to the broad conception of “learning healthcare systems” (Budrionis and Bellika 2016; Faden et al. 2013). In short, this translational genomic medicine (TGM) agenda is about integrating the organization of clinical and preclinical research with routine healthcare, to better reap alleged benefits of new advances in big data, gene technology, and genomic research. This paper considers this agenda (further specified below) from an ethical standpoint, with particular concentration on justifiable resource allocation.

Many have observed that a translational organization of medicine threatens to blur the practical boundary between clinical ethics and research ethics, and therefore requires additional safeguards and ethical oversight mechanisms; and powerful business interests in the background have been held out as one particular challenge for justified resource allocation (Bærøe 2014; Cribb 2010; Hostiuc et al. 2016; López de la Vieja 2016). In addition to this, this paper adds the observation that the issue of resource allocation, while having to take clinical or research ethical standards into account, becomes differently shaped depending on whether or not TGM is perceived as clinical routine or research. Based on two distinct models of what the rationale of TGM may amount to, it is argued that TGM faces a dilemma between upholding clinical ethical standards and having a good chance of ethically justifying the costs required for TGM. Further ethical challenges—in general as well as to justified resource allocation to TGM—emerge when the two models are confused or mixed. Based on this, it is proposed that TGM should presently be viewed as a pure research endeavor, where entire treatment concepts of TGM are evaluated, and that resource allocation to the area should be ethically assessed on this basis. While justified allocation of resources requires a sound design from a research ethical perspective, it also needs to be compared to the ethical benefits and downsides to alternative health research investments.

## General assumption

The paper makes use of some elementary assumptions about TGM and regarding ethical justification of research as well as clinical practice. First, TGM is taken to mean a way of organizing genomic research and clinical practice, so that genomic research facilities and activities are used and applied in immediate interaction with clinical healthcare. These facilities usually include advanced sequencing, analysis against research databases to increase understanding, and (sometimes, and increasingly) the design and evaluation of experimental treatments. Conditions in focus potentially include anyone with a significant genetic component (more or less hereditary, expressive, rare, or complex), and any condition where genetic approaches to treatment (such as pharmacogenetic cancer care, or stem cell or genetic modification therapy) may be considered.

Second, it is assumed that establishing a TGM operation incurs massive costs (further detailed below) that need to be justified by benefits (reasonably) balancing the opportunity costs, i.e., the benefits in other areas that are lost due to this resource allocation. The benefits of TGM need to be *worth* these lost benefits in order for this allocation to be justified (Lomas et al. 2018). To make this justification, health economical perspectives are helpful, but also ethical principles are needed, elaborated in the fifth point below.

Third, what is seen as a benefit can vary, and does vary, between medical research and clinical medicine. In the latter case, the benefits are determined by the level of health improvement for treated patients, in the former by the contribution to the advancement of scientific knowledge. It is well known that these two aims may conflict in a variety of ways, and this is the reason behind research ethical consensus documents, such as the Declaration of Helsinki (WMA 2013), setting limits for what may be done to patients, and on what conditions, in the name of scientific advancement. A recent international research ethical guideline instead talks about the “social value” of research, where the aims of scientific advancement and of health improvement are mixed (CIOMS 2016). However, within that conception, the two aims will still need to be balanced in cases of conflict.

Fourth, balancing the benefits and opportunity costs of TGM requires sufficient evidence to back up assessments, and also here, standards vary between research and clinical practice. In the latter case, standards are usually higher, while more of uncertainty is accepted to continue to pursue a research hypothesis in a scientific context. This difference follows from the fact that research and clinical practice pursue different ends.

Fifth, ethical reasons are crucial for deciding what counts as benefits and risks, how these are to be balanced, how standards of evidence are set, and how costs and opportunity costs are to be compared. For instance, in orphan disease policy schemes, contested ethical reasons of fairness and harm-reduction are often used to motivate spending resources on treatments, in spite of disproportionate pricing, weak evidence, and uncertain benefits (Juth 2017; Rodriguez-Monguio et al. 2017).

## Two models of justifying TGM

The TGM agenda can be implemented according to two distinct models for how it is motivated and justified.^1^ In practice, both models may be mixed in a TGM setup, but the differences between them for motivating and justifying the practice will still persist.

First, there is what is here termed the *Lab Assisting Clinic-model* (LAC), where research facilities and resources are supposed to enhance the clinical diagnosis and treatment of patients. The context may be one of a single patient in non-standard circumstances, where diagnosis and/or effective treatment cannot be achieved within available clinical routine, as is often the case with rare genetic conditions. Or the context may be one where known general uncertainties are managed with a personalized or precision medicine routine (e.g., in cancer treatment or in the management of more common genetic syndromes with variable expression), and research facilities and resources are employed as a part of that. In both cases, the model means that clinical questions from the healthcare organization are put to the research organization, and answers returned from the latter are supposed to enhance the quality of care. Both these types of the LAC model of TGM (sometimes advertised as “clinical genomics”) are being implemented at an increasing number of universities and research hospitals around the world.^2^

Second, there is the Clinic Assisting Lab-model (CAL), where the direction of queries and answers which may motivate the practice goes in the opposite direction to LAC. That is, clinical practice is used as a resource for research in this context, with scientific facilities and expertise operating in close proximity to routine healthcare, enabling swift transitions between the role of a person as a patient and as a research subject. Again, the context may be to identify rare extraordinary individuals (suffering from genetic or conditions, or conditions otherwise relevant from the perspective of genomics, that are difficult to treat or research in other ways), suitable for opportunistic data-gathering and experimentation. Or it may be a large-scale trial operation designed to trawl clinical findings for information of interest from a scientific standpoint, often with the plan of having data be fed into a “big data” operation. Genetic screening programs in the neonatal and prenatal area are nowadays often organized in this manner, to feed into bio- or databanks that can in turn be utilized for research.

As mentioned, these justifying rationales are often mixed in the TGM practice, albeit emphasis in the experience of different practitioners or in public presentations in different contexts may be more on the one than on the other. In reality, the rationale and basis for justifying actual TGM operations therefore often seem rather fuzzy with regard to which of these two models set out the rationale that justifies the practice (see the webpages listed in footnote 2, for instance). In effect, very different rationales (to serve the interests of particular patients or to serve the interests of science or of society in general) are regularly mixed or confused, resulting in potential ethical problems. This is illustrated by a number of scandals. One type regards unethical data gathering and sharing, illustrated by the *care.data* initiative in the UK (Godlee 2016), and a recent similar attempt at Karolinska Institutet and the Karolinska University Hospital in Sweden to hoard patient data to illegally move it abroad to the private big data company *ICHOM* (both these operations are now defunct) (Andersson 2017; Cederberg 2018). Another family of scandals is about experimental treatment on patients without ethical permission and oversight, such as in the infamous case of Paolo Macchiarini (also including serious research misconduct, besides blatant ethical breaches) (Hawkes 2018; Karolinska Institutet 2018), and several similar (albeit less spectacular and widely publicized) events within regenerative medicine surgery at universities in the UK, Germany, and Sweden^3^. Contacts with representatives of more serious translational operations convey that they too usually have a mixed agenda, without clear procedures for determining when one or the other aim is the target of decisions. This has regarded LAC operations at specific clinics that express hope for scientific advances in the slipstream of a TGM operation (again, see footnote 2). But also CAL organizations, such as the *100K Genomes* project in the UK and several biobank initiatives, mix research aims with expressions about prospects for clinical benefits in advocacy and public presentations. In short, TGM in practice often seems to be aiming at moving both ways between the “bench” and the “bedside,” but at the same time be unclear about how these directions are mixed when attempting to justify the practice, and how shifting ethical standards are handled.^4^

## Ethical resource allocation parameters through the LAC lens

The costs of establishing a TGM approach are considerable. They are accrued by securing and maintaining the required infrastructure (labs, computers, new buildings, etc.) and working materials (for lab work and data analytics), house appropriate competence, keep a surrounding organization to make the TGM-operation work, and so on. What benefit would be achieved from the point of view of the LAC model that could have a potential for justifying these costs?

The main benefit held out when the LAC model is used to motivate TGM is better diagnosis. However, diagnosis is not a health benefit of the sort required to justify costs for clinical practice. In order to have such a benefit, the improved diagnosis has to be linked to interventions depending on this diagnosis, which in turn makes patients sufficiently better off. However, this introduces a general problem of evidence. The strategy to have research facilities provide special solutions for very rare patients in extraordinary circumstances makes it very difficult to have any volume of similar data to analyze the actual effect of interventions, and the concept itself excludes the presence of anything like a control group. At best, there will be tiny (but perhaps many) series of very rare health problems, where the outcome of a TGM strategy might be compared to historical controls (if previous documentation can provide sufficient details). When the situation for acquiring good evidence is as poor as this, it is sometimes argued that if a condition is very serious and lacks an effective existing treatment (as in the case of many rare genetic conditions), it may nevertheless be clinically ethically defensible to test unproven interventions. This kind of thinking has inspired the idea of a specific “learning healthcare systems” ethics and, in extension, the notion of a “right to try” experimental treatments (Bateman-House et al. 2015; Faden et al. 2013). However, the strength of a right of doctors to experiment with their patients or a right to try has to be assessed in light of the very real risk making already very burdened patients not only feel cheated (along with family and friends) but much *worse* off due to unforeseen side-effects (Dresser 2015). The underlying problem of evidence repeats itself when we consider more large-scale TGM operations in the form of personalized or precision medicine strategies through the lens of the LAC model. Such approaches necessarily mean that each group of patients identified with a very specific variant of a disease and therefore exposed to a very specific intervention will be quite small, and if the patients also suffer severe symptoms, this makes for a challenging situation for anyone trying to prove effect and safety (El-Alti et al. 2019). There may be indications of minor, albeit rather uncertain, effects, but usually (as in many cases of cancer treatments introduced during the last decade) accompanied by significant side-effects that further undermine the prospect of actual net benefit (Banzi et al. 2015; Cohen 2017), or (as in the case of some recent interventions for hitherto untreatable genetic conditions, such as spinal muscle atrophy) lingering uncertainty of the actual long-term effectiveness, and possible future downsides. In any case, it is difficult to get around the fact that TGM operations assessed from the LAC model typically imply experimenting on especially vulnerable patients with very uncertain prospects and rather significant risks.

A more promising prospect for demonstrating effects of sufficient magnitude to justify TGM costs through the LAC lens may appear by shifting perspective from specific, individual treatments and instead consider the entire *concept of TGM*. However, at this time, we are far from having any kind of proof regarding the extent to which TGM produces a sufficient improvement of patient benefit compared to previous diagnosis and treatment strategies in order to justify its costs from a LAC perspective. To produce such proof, it would seem necessary to move the basis of assessment from the LAC model and instead design studies where the TGM concept can be compared over a sufficient time to alternative treatment concepts that do not accrue the cost increases implied by TGM. Rather than LAC, this would move the framework of justifying TGM opportunity costs from the ethics of clinical practice and into the research ethics context of the CAL model (see next section). However, also considering this prospect, it must be remembered that the justification of the costs of the entire concept of TGM through the lens of LAC needs to be in the form of benefits of this concept in comparison to what we would get out of the resources if these were instead channeled to other patient groups where effects are easier to attain and prove. So, even if TGM as a concept could be proven to be an *improvement* based on LAC, it is an open question if that justifies reserving the money for this particular improvement, rather than improvements for other parts of healthcare.

## Ethical resource allocation parameters through the CAL lens

The CAL model of TGM is no different from the LAC model with regard to the costs that need to be balanced by sufficient benefits to justify opportunity costs. The difference lies in what type of benefits of TGM the rationale of CAL holds out. As we are here evaluating not a clinical resource investment, but a research investment, the relevant benefits are ones of relevance to the evaluation of research. As noted, this does not necessarily exclude paying attention to (likely) health benefits (if any can be substantiated) but is nevertheless focused on advancement of medical scientific knowledge (rather than the immediate health of research subjects) as the rationale that is supposed to justify opportunity costs.

At the same time, the CAL model for justifying TGM builds on the idea of exploiting clinical medical resources in the forms of patients for research purposes. This, of course, actualizes well-known tensions between research aims and clinical ethical standards, as well as acute risk of major breaches of widely acknowledged clinical and research ethical boundaries. This is especially obvious when the CAL rationale for TGM (as in the case of Macchiarini and similar setups) is discharged in the form of organized opportunistic experimentation on individual patients in exceptionally vulnerable circumstances. But the basic ethical hazards remain the same also when the form is that of a large-scale routine experimentation operation to test some precision medical strategy. First of all, having research so intimately integrated with the clinical practice drastically increases the risk of *therapeutic misconception*; i.e., patients and/or professionals do not clearly realize what actions are proven clinical practice and what actions are unproven and experimental, made primarily for the purpose of advancing science (Appelbaum et al. 1987; Appelbaum et al. 2004; Appelbaum et al. 2012). Second, running TGM motivated from the CAL model as a clinical routine setup would similarly violate what has been called the *research exceptionalism* default of clinical medical ethics, i.e., the idea that patients should not be viewed as guinea pigs for scientific purposes and that clinical research therefore require special justification and arrangements (Wilson and Hunter 2010). Third, specifically, informed consent standards (typically more demanding for research than for clinical practice) are more likely to be violated when a CAL rationale motivates an operation within the confines of a clinical practice routine. Fourth, similarly, such setups will likely expose patients to clinical uncertainties and risks that are otherwise viewed as unacceptable from a clinical ethical perspective.

As indicated at the end of the previous section, there is one way forward to respond to these challenges, namely, to run *bona fide* trials to evaluate entire TGM *concepts* (designed to target suitable patient populations). It may be discussed to what extent such trials must be controlled, but I would argue that historical controls must at the very least be a part of such an evaluation and that the risk of exposing large amounts of very vulnerable patients to possible downsides is an important argument for genuine RCT designs^5^. Such trials must then, of course, be reviewed in the ordinary fashion to establish that they are, indeed, ethically justified (based on the CAL model). First, one needs to determine whether or not there is a genuine clinical equipoise motivating the need for a trial from a clinical ethics perspective (justifying that some patients are deprived of whatever is the current standard procedure). Second, research protocols and procedures must ensure that research ethical standards with regard to safety and patient autonomy are met and avoiding the pitfalls mentioned above.

None of this will change the basic resource allocation ethical challenge for TGM to justify its (opportunity) costs. As in all research, the benefits of investing in TGM will remain unclear until the results of the trials are in. Potentially, there are, of course, huge scientific benefits of the TGM concept. However, it remains unclear if these prospects of scientific advances are good enough to motivate the spending of research resources in this area, rather than some other area of science (say, antibiotic development, astrophysics, or climate science). To tackle this objection, TGM advocates may point to a particular importance for society (e.g., stressing the increased relative importance of controlling genetic effects in medical treatment as other effects have already been controlled). However, to demonstrate benefits of TGM to underpin such a claim, TGM will need to not only show good prospects for the advancement of scientific knowledge but also that this advancement will, in fact, bring sufficiently likely and important (public) health benefits. In terms of the recent CIOMS guidelines for health-related research ethics, *social* and not only scientific value must be demonstrated (Wendler and Rid 2017). Again, it remains to be seen if such prospects are viable enough to justify the opportunity costs for other areas of (medical) science accrued when massive resources are concentrated to TGM projects.

## Concluding discussion

To justify the allocation of resources to a particular area of medicine, it is necessary to justify its opportunity costs, appealing to some rationale linked to ethical principles. Based on the investments involved (described earlier), these costs can be expected to be considerable in the case of TGM. The LAC model does not necessarily imply that TGM violates clinical or research ethical tenets, but since the benefits of TGM remain to be demonstrated, it is difficult to justify its (opportunity) costs. The CAL model moves closer to providing justification for allocating resources to TGM, but at the expense of severe clinical and research ethical hazard. This hazard can be avoided if TGM justified by the CAL model is organized in the form of controlled trials of *entire TGM concepts* and assessed and reviewed according to regular research ethical standards and procedures.

In many current TGM operations, it remains unclear to what extent the rationale is that of LAC or that of CAL, or if it is both in an unclear mix. This gives rise to further ethical uncertainties, visible in recent scandals in the regenerative medical and big data health research areas. As suggested frameworks for translational research ethical oversight build on the basic notion of clearly identified boundaries between different aims (Bærøe 2014), this further undermines the prospect of justifying TGM from a resource allocation standpoint.

Both of these hurdles might be overcome. TGM initiatives may be coordinated into larger *concept trials* for large groups of patients with different types of problems (all suitable for a specific general TGM pathway), and the ethical downsides coming from unclear aims may be avoided by being crystal clear to patients and society that this is experimental clinical research (testing a new diagnosis and treatment concept). Such trials may be approved through standard routes for research ethical oversight to the extent that clinical equipoise can be substantiated and standard requirement of risk-harm balance and informed consent are met. The reasoning to support approval of such trials may mention lack of existing effective diagnostic and therapeutic interventions for the patient group targeted (to the extent that this is so), the importance of testing new treatment concepts for groups that have been unfairly overlooked in past research (to the extent that this is so), the possible benefit of assembling large repositories of information to address the needs of patients in these groups (provided that there is such a benefit), and—of course—the severity of the conditions found in the patient group.

However, this will still leave open to what extent the (often massive) investment in TGM can be ethically justified in view of its (opportunity) costs. Here, the arguments for approving TGM concept trials may, of course, be reused. Especially the fairness and need related reasons may add fuel to this effect, to the extent that the TGM concept trial targets a patient group with such features. But none of this will guarantee that the resources invested could not have been better and more fairly used elsewhere, in areas with similarly ethically strong features. To make this assessment, at the very least, a more complete map of competing alternative health infrastructure investments would need to be charted. On such a basis, a combined ethical and health economic analysis could be made, to ascertain whether or not the investment in TGM infrastructure is justified. But without such a map, analysis to this effect remains impossible. Another route may be to compare to *past* health infrastructure investment decisions, which often have been made on the basis of unclear overall cost-benefit justification profiles. However, such reasoning is open to the obvious objection that these past investments might very well have been unjustified themselves.

## Footnotes

1 Of these, LAC more or less equals the “bench to bedside” slogan in the TGM literature. CAL, I have never encountered explicitly described as an approach in the literature, but it is clear that the model exists from observations of what has actually been going on regarding TGM setups, as well as from conversations with TGM practitioners and advocates. Some examples are provided later in the text. The terminology has been chosen to focus on what is of ethical importance (regarding what aims are pursued and how in TGM), rather than to evoke lively images of labs, clinics, health professionals, and patients that may distort the ethical assessment.

2 For some examples, see the following: http://www.chp.edu/our-services/rare-disease-therapy https://www.med.lu.se/ctg https://www.tgen.org/ https://www.mayo.edu/research/departments-divisions/department-clinical-genomics-research https://wcmtm.gu.se/research-groups/genomics-platform http://www.translational-genomics.uni-koeln.de/ https://keck.usc.edu/translational-genomics/ https://research.qut.edu.au/translationalgenomicsgroup/ https://www.ucl.ac.uk/cardiovascular/research/population-science-and-experimental-medicine/centre-translational-genomics https://dceg.cancer.gov/about/organization/programs-hgp/ltg https://www.helmholtz-muenchen.de/itg/index.html

3 Comprehensively documented and reported by German independent investigative science journalist Leonid Schneider: https://forbetterscience.com/?s=Trachea

4 The evidence here is unsystematic, anecdotal, and selective, albeit the *100K Genomes* observation was confirmed by leading representatives of that project at the symposium leading to the publication of the present special issue. The examples provided in note 2 above also convey mixed messages with regard to if the aim is healthcare or research or something in between. More systematic empirical investigation into the possible mix of clinical and research agendas within TGM would be beneficial for assessing the volume of this potential ethical problem.

5 The controls are, of course, essential for the trial to be able to demonstrate anything with regard to effect and safety of TGM. How such controls are accomplished in a trial may vary depending on the context, but given that we talk about an overarching diagnosis and treatment concept for a large variety of conditions and not trials of a single intervention for a single conditions, a multicentral model, where some are intervention centers and some are control centers, seems like an a priori plausible notion.
